# Cochlear Aqueduct Morphology in Superior Canal Dehiscence Syndrome

**DOI:** 10.3390/audiolres13030032

**Published:** 2023-05-15

**Authors:** Nimesh V. Nagururu, Diane Jung, Ferdinand Hui, Monica S. Pearl, John P. Carey, Bryan K. Ward

**Affiliations:** 1 Department of Otolaryngology—Head and Neck Surgery, Johns Hopkins University School of Medicine, Baltimore, MD 21205, USA; 2 Department of Radiology, Johns Hopkins University School of Medicine, Baltimore, MD 21205, USA; 3 Department of Radiology, Children’s National Hospital, Washington, DC 20010, USA

**Keywords:** cochlear aqueduct, superior canal dehiscence syndrome, flat-panel CT

## Abstract

The cochlear aqueduct (CA) connects the scala tympani to the subarachnoid space and is thought to assist in pressure regulation of perilymph in normal ears, however, its role and variation in inner ear pathology, such as in superior canal dehiscence syndrome (SCDS), is unknown. This retrospective radiographic investigation compared CA measurements and classification, as measured on flat-panel computerized tomography, among three groups of ears: controls, *n* = 64; anatomic superior canal dehiscence without symptoms (SCD), *n* = 28; and SCDS, *n* = 64. We found that in a multinomial logistic regression adjusted for age, sex, and BMI, an increase in CA length by 1 mm was associated with a lower odds for being in the SCDS group vs. control (Odds ratio 0.760 *p* = 0.005). Hierarchical clustering of continuous CA measures revealed a cluster with small CAs and a cluster with large CAs. Another multinomial logistic regression adjusted for the aforementioned clinical covariates showed an odds ratio of 2.97 for SCDS in the small CA cluster as compared to the large (*p* = 0.004). Further, no significant association was observed between SCDS symptomatology—vestibular and/or auditory symptoms—and CA structure in SCDS ears. The findings of this study lend support to the hypothesis that SCDS has a congenital etiology.

## 1. Introduction

The cochlear aqueduct (CA) is a small bony channel of the otic capsule connecting the scala tympani adjacent to the round window to the subarachnoid space of the posterior cranial fossa [1]. The function of the CA is not well characterized, but the duct is thought to act as a pressure-relief valve normalizing the pressure differential between the perilymph and CSF [2]. In normal ears, it may filter fluctuations in cerebrospinal fluid pressure associated with cardiovascular dynamics and breathing and may contribute bone-conducted sound at lower frequencies [3,4]. However, the contribution of CA dynamics in the context of inner ear disease is unknown.

In superior canal dehiscence syndrome (SCDS), an opening of the bony labyrinth overlying the superior semicircular canal is thought to act as a pathologic third mobile window disrupting normal sound-vibration transmission between the oval and round windows [5,6]. The abnormal shunting of acoustic energy into and out of the vestibule by way of the dehiscence produces a constellation of characteristic symptoms including sound- and pressure-induced vertigo/oscillopsia, autophony, bone conduction hyperacusis and pulsatile tinnitus [6,7]. The consensus definition of SCDS diagnostic criteria requires the aforementioned symptoms along with evidence of dehiscence on high-resolution CT and physiologic signs or testing consistent with SCDS [8].

While the past two decades have seen an abundance of research characterizing SCDS, several questions remain unanswered. Interestingly, not all individuals with anatomic superior canal dehiscence (SCD) have SCDS-characteristic symptoms and testing. One temporal bone histopathology study found the prevalence of SCD to be 0.7% in the general population, whereas the prevalence of SCDS, while unknown, is likely far less [9]. Similarly, the etiology of SCDS is unclear. The majority of SCDS presentations occur in adulthood, yet the disease is thought to be congenital as a thin or dehiscent bone over the superior canal is often bilateral. In some patients, the onset of SCDS symptoms is thought to be due to an insult such as a head injury or barotrauma, perhaps on top of congenital predisposition [10]. Additionally, symptom presentation is diverse; individuals may present with either auditory or vestibular manifestations alone or both simultaneously, and our understanding of why is limited.

Given that the CA is a physiologic window of the vestibule and plays a role in pressure management in the inner ear, we hypothesize that variations in CA morphology may be associated with the development of SCDS and the symptoms patients report. The pressure transmission through the CA in early development could lead to aberrant development of the otic capsule and the dehiscence seen in SCDS or perhaps increased pressure transmission through an enlarged CA is more likely to result in symptoms of SCDS in individuals with a congenitally dehiscent superior semicircular canal. In this investigation, we use high-resolution flat-panel computerized tomography (CT) to characterize the CA with respect to superior canal pathology status and symptomatic presentation.

## 2. Materials and Methods

### 2.1. Study Population and Data Source

Individuals over 18 years old who received a flat-panel CT scan without contrast for otologic indications between January 2018 and May 2022 at our tertiary-level care center were eligible for the study. Both temporal bones of these individuals were treated as independent samples. Bones were excluded from this investigation if their scans contained artifacts or showed evidence of anatomic disruption from disorders including otosclerosis, labyrinthitis ossificans, and fracture. After filtering the data set for inclusion and exclusion criteria, common indications for obtaining the study included investigation for suspected SCDS, pulsatile tinnitus, conductive hearing loss, and cholesteatoma. Flat-panel CT scans (DynaCT; Siemens, Erlangen, Germany) were conducted using previously described protocols yielding resolutions as small as 0.1 mm in bony structures [11]. Demographics, clinical presentation, and physiologic tests were extracted from the electronic medical record. Approval for this investigation was granted by the Johns Hopkins Institutional Review Board under IRB00279939.

### 2.2. Superior Canal Pathology and SCDS Symptom Classification

Based on the radiology report and patient history, ears were divided into three groups: control, SCD, and SCDS. Ears in the control group lacked any evidence of anatomic superior canal dehiscence in their imaging. Ears in the SCD group showed anatomic dehiscence of the superior semicircular canal but did not meet the Bárány Society criteria for superior canal dehiscence syndrome [8]. Ears in the SCDS group had both anatomic dehiscences as well as the physiologic testing and clinical symptoms needed to diagnose SCDS. A power analysis for a two-tailed pairwise comparison of groups, with a power of 0.8 and Cohen’s D of 0.5, suggested that 64 ears should be included in each group. Hence, 64 ears were selected, if available, randomly from each group out of all eligible ears in the database. Ears in the SCDS group were identified as having auditory manifestations if they presented with bone-conduction hyperacusis (i.e., hearing internal sounds abnormally loud in the affected ear) or pulsatile tinnitus and vestibular manifestations if they presented with time-locked, sound- or pressure-induced vertigo.

### 2.3. Cochlear Aqueduct Morphology

**Figure 1:**
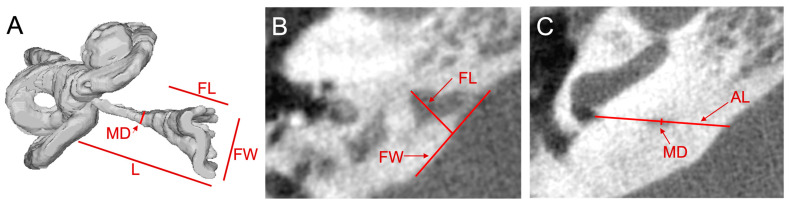
Cochlear aqueduct measurements of right ear on flat-panel CT scan. (**A**) 3D reconstruction of an inner ear showing the location of measurements of the cochlear aqueduct (CA) recorded from the axial slices shown in *B* and *C*. Midpoint diameter (MD), length (L), and medial aperture funnel length (FL) and funnel width (FW) are labeled. (**B**) Axial slice at the level of the greatest funnel width labeled with FL and FW which are measured in this image. (**C**) Axial slice at the level of the CA’s opening into the inner ear near the basal turn of the cochlea; MD and axial length (AL), or length of CA in the axial slice, are labeled.

Cochlear aqueduct dimensions were measured for each ear in the axial plane using Philips Carestream Vue PACS (version 12.2.6.304). Since the CA traverses many axial slices, CA measurements were made using different slices. CA measurements were adapted from the protocol described by Wichova and colleagues and are shown in Figure 1A [12]. The CA has a funnel shape with a wider opening at the medial aperture that narrows near its midpoint before entering the cochlea at its lateral aperture. As shown in Figure 1B, the funnel width at the medial aperture was measured in the axial slice with the largest funnel width. The funnel length is the measurement between the funnel width at its midpoint and the narrowest part of the funnel. The midpoint diameter of the CA was measured in the slice in which the CA was approximately halfway along its length in the axial slice as is seen in Figure 1C. The length of the CA was not typically visible in a single axial slice but, rather, had to be calculated over several slices using the Pythagorean theorem. Considering the length of the CA as the hypotenuse of a right triangle, one leg of the triangle was measured as the distance between the lateral and medial apertures of the CA in the slice in which the cochlear or lateral aperture was visible; the measurement of this leg is seen in Figure 1C. The second leg was the z-stack distance between the cranial (medial) and cochlear (lateral) aperture which was calculated by multiplying the number of slices between the slice in which the apertures were visible and the slice thickness. The length of the CA was the hypotenuse of these two legs, calculated using the Pythagorean theorem. The course of the CA on flat-panel CT is shown in Video S1 in the supplement.

The CAs were also classified according to the schema previously defined by Migirov and Kronenberg [13]. Type 1 CAs are visualized, at minimum as a streak, along their entire course. Type 2 CAs are visible in the medial two-thirds of their course but are not completely visualized entirely in the lateral third. Type 3 CAs are not visualized completely along the medial two-thirds of their course. Type 4 CAs are undetectable or unidentifiable. Authors NVN and BKW established appropriate use of the CA morphology assessment protocol together, and, blinded to groupings, author NVN completed CA measurements and classification for all ears.

### 2.4. Statistical Methods

All variables were tested for normality using the Shapiro-Wilk test to select the appropriate parametric or non-parametric test. Statistical tests included Pearson’s chi-squared test, the Kruskal-Wallis rank sum test, Fischer’s exact test, and analysis of variance [14,15,16,17,18]. Demographics were calculated for the overall study population and then compared among the three groups. Three primary analyses of outcome variables were performed. First, CA measurements and classification were compared among normal, SCD, and SCDS groups. Second, for ears with SCDS, CA measurements were compared between auditory and vestibular symptom groups. Third, because multiple measurements of the CA were recorded, clustering was used to group ears based on the similarity of their cochlear aqueducts to identify distinct phenotypes. Hierarchical clustering (Euclidean, complete-linkage) was performed using the measurements of CA length, midpoint diameter, funnel length, and funnel width. This analysis separated CAs into two clusters [19]. Demographics and incidence of superior canal pathology were then compared between clusters. Similar hierarchical clustering was done for CAs in ears with SCDS, and SCDS symptoms were compared between the resulting clusters. All significant omnibus tests of outcome variables underwent post hoc pairwise analysis; *p*-values in the pairwise analysis were adjusted with the Benjaimini–Hochberg method to avoid type I errors from performing multiple comparisons [20]. Significant findings then underwent multinomial logistic regression with clinically significant covariates [21]. All analyses were conducted in RStudio (Version 2022.07.2) with a significance level of 0.05.

## 3. Results

### 3.1. Demographics

A total of 602 temporal bones were included after applying inclusion and exclusion criteria of which 370 were classified as controls, 28 as SCD, and 87 as SCDS. After random sampling, there were 64, 28, and 64 ears in the control, SCD, and SCDS groups respectively. Ears in the study population belonged to individuals with a mean age of 49.5 (SD: 13.8) years old, 61% female, and 90% white race (Table 1). The median BMI of these individuals was 26.9 (Interquartile Range (IQR): 22.6, 31.1) kg/m^2^. Race was the only demographic significantly different amongst the control, SCD, and SCDS groups (80%, 93%, and 98% white respectively; *p* = 0.009).

### 3.2. CA Morphology and Superior Canal Pathology Classification

Out of 156 total ears in the analysis, 96 or 61.5% had CAs classified as type 1, or visible along their entire course (Table 2). Funnel length, funnel width, midpoint diameter, and CA classification were not significantly different among study groups on omnibus testing. Mean CA length was significantly different among control, SCD, and SCDS groups (13.35 mm, 12.97 mm, 12.32 mm; *p* = 0.018). Post-hoc testing of CA length showed a significant difference between the control and SCDS groups (*p*-adj = 0.014). A multinomial logistic regression model for SCDS (with the control group as reference) and CA length, adjusted for age, sex, and BMI, showed a persistent difference in CA length between SCDS and control groups. A one-millimeter increase in CA length was associated with an odds ratio of 0.760 for being in the SCDS group vs. the control group (95% confidence interval (CI): 0.627, 0.920; *p* = 0.005).

### 3.3. CA Morphology and SCDS Symptoms in SCDS Ears

Table 3 shows cochlear aqueduct measurements for SCDS ears separated by symptomatic manifestation. Vestibular and auditory SCDS symptoms were seen in 49/64 (76.6%) of SCDS ears, and auditory symptoms alone in 15/64 (23.4%). Vestibular symptoms alone were not seen in any ears. Neither demographic nor CA variables or classification were significantly different between symptom groups.

### 3.4. Clustering of Ears by CA Measurements

**Figure 2:**
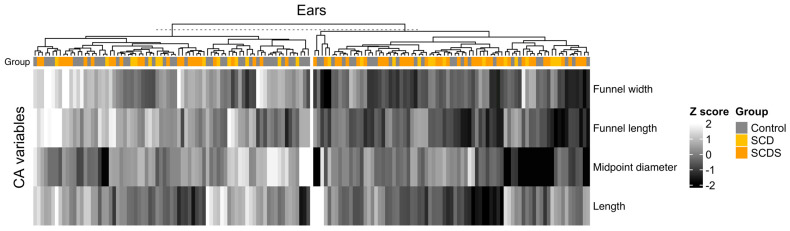
Hierarchical clustering of all ears by cochlear aqueduct measurement. Individual ears are represented by columns. K-means-based hierarchical clustering was then used to classify ears into two clusters using the four CA variables as shown by the tree diagram. Superior canal pathology grouping for each ear is annotated above clusters. Ears with Type 4 CAs were removed from clustering (one in control, one in the SCDS group). Cluster 1 is the group of CAs on the right and Cluster 2 is the group on the left.

Figure 2 shows the hierarchical clustering of cochlear aqueducts annotated with group classification. Demographics, CA measurements, and superior canal pathology classification are shown in Table 4. Demographics were similar between clusters. CAs in cluster 1 had narrower (4.37 mm vs. 5.90 mm) and shorter funnels (3.98 mm vs. 5.37 mm), smaller midpoint diameters (0.29 mm vs. 0.41 mm), and shorter lengths (11.94 vs. 13.65 mm) as compared to cluster 2. Omnibus testing showed a difference in the frequency of superior canal pathology groups between clusters (*p* = 0.017). Post-hoc testing showed fewer control ears (30% vs. 51%, *p*-adj = 0.024) and more SCDS ears (52% vs. 31%, *p*-adj = 0.027) in cluster 1 as compared to cluster 2. In multinomial modeling after adjusting for age, sex, and BMI, the odds of being in the SCDS group for cluster 1 are 2.97 compared to cluster 2 (95% CI: 1.31, 6.75; *p* = 0.004). When SCDS ears were clustered further by CA measurements, no significant association was found between clusters and SCDS symptomatology. Figure S1 shows the heatmap for the clustering of SCDS ears by CA measurements, and Table S1 shows demographics, CA measurements, and SCDS symptomatology for SCDS ears by the clustering.

## 4. Discussion

This is the first investigation of CA morphology in ears with SCDS. Our objective was to probe the association between the CA and SCDS by evaluating CA measurements in control ears, ears with SCD alone, and ears with SCDS, with the goal of understanding whether there is an association between duct morphology and the diagnosis of SCDS. We also sought to investigate whether the structure of the CA was associated with the symptomatic, vestibular, and/or auditory, presentation of SCDS.

**Figure 3:**
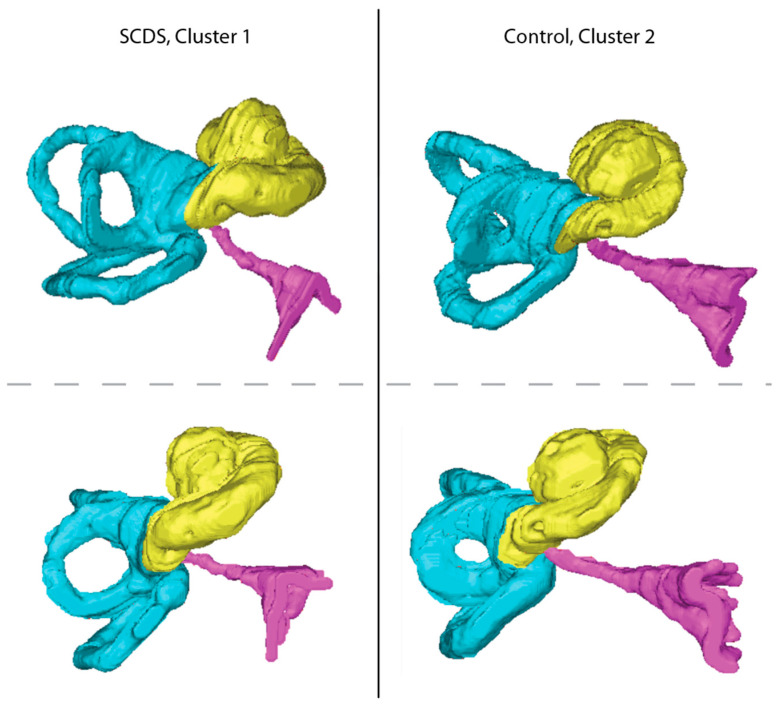
Representative ears from the clusters produced by hierarchical clustering. The left panel shows two inferior perspectives of a representative ear with SCDS and a CA from cluster 1. The right panel shows two inferior perspectives of a representative ear of a Control from cluster 2. Note the smaller size of the cochlear aqueduct in the cluster 1 ear as compared to that of the cluster 2 ear.

We found an association between superior canal pathology and cochlear aqueduct structure. In the individual evaluation of CA measurements, CA length was significantly shorter in the SCDS group than in the control group. Hierarchical clustering based on the four CA measurements allowed for holistic consideration of the structure. Cluster 1, characterized by ears with smaller CA measurements in all dimensions, was found to have a greater prevalence of SCDS compared to cluster 2 with an odds ratio of 2.97. 3D reconstructions of representative ears and their CAs from each cluster are shown in Figure 3.

When the CA is modeled as allowing flow, it is often modeled as a simple patent tube. In these models, impedance, and flow inversely and directly vary, respectively, with the fourth power of the diameter as dictated by the Poiseuille equation. Given the contribution of CA diameter, smaller CAs as found in Cluster 1 may lead to inappropriate pressure regulation in the inner ear, especially in early life when CAs are wider and more free of arachnoid mesh, and contribute to the aberrant development of the bone overlying the superior semicircular canal [22]. In adults, however, the impedance of the cochlear aqueduct is already relatively large, and intracranial pressure contributions reach the inner ear, largely, by way of the vestibular aqueduct; hence an increase would likely result in negligibly different fluid dynamics [23,24]. Moreover, the cochlear aqueduct was found to be patent only in 34% of temporal bones in one histopathologic study [3]. More likely, the association between smaller CAs and SCDS is not causal but rather confounded by generally smaller temporal bones in individuals with SCDS. If CA length is considered a proxy for temporal bone size, the finding of shorter CAs in this study may lend support to the congenital hypothesis of SCDS etiology. For a relatively fixed size of the otic capsule, the development of a smaller surrounding temporal bone would more likely result in the apex of the superior semicircular canal being insufficiently covered in bone. This could result in SCDS in childhood, but this is rare [10]. More often symptoms may not manifest in childhood, perhaps because the thin endosteal bone of the otic capsule or stiff dura still prevents pressure transmission early in life. However, the deficit of overlying bone may predispose to symptomatic presentation after a second insult like head trauma or barotrauma [25].

The SCD group or group with anatomic dehiscence without syndromic presentation showed no significant differences in CA measurements as compared to both SCDS and control groups. However, CA length and midpoint diameter for the SCD group did fall in between those of the SCDS and control groups, suggesting that the aforementioned explanations of the relationship between SCDS and CA may be at play to a lesser extent with SCD ears. The lack of significance in this finding may be due to the small sample size of SCD ears.

No significant association was identified between SCDS symptomatology and CA morphology in SCDS ears in the cluster analysis. As the biomechanical basis of both auditory and vestibular manifestations of SCDS overlap, it is poorly understood why some individuals with SCDS lack either auditory or vestibular symptoms [26,27]. Researchers have postulated that the cochlear aqueduct may be an explanation for this phenomenon of variable symptomatology [28,29]. However, our data do not support this hypothesis. The impedance of superior canal dehiscence is likely vastly lower than that of the CA under any circumstances, even if the CA is patent, because of the long and thin nature of the CA compared with the extremely short distance across canal dehiscence and the much larger diameter of the typical dehiscence (several mm) vs. CA midpoint diameter (median 0.35 mm, Table 2). Thus, the disruption in the pressure balance between the endolymph and perilymph contributing to abnormal acoustic energy transmission in SCDS are likely governed by parameters of the dehiscence and not by features of the CA [27]. Other works have also explored the contributions to the variable presentations of SCDS from variations in round window compliance, dehiscence size, dehiscence location, and differential central compensatory mechanisms [30,31,32].

**Figure 4:**
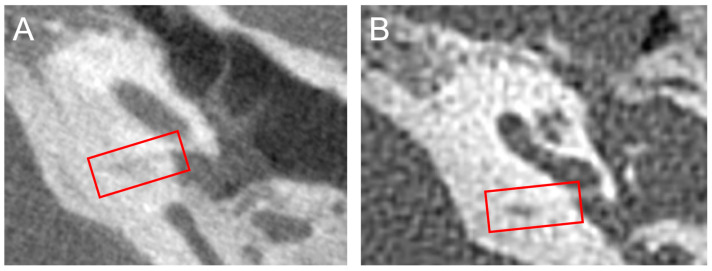
Side-by-side flat-panel and high-resolution computerized tomography of the same patient. (**A**) Flat panel CT scan (parameters described in the Methods section) demonstrating the improved resolution of the imaging modality as compared to the high-resolution CT. A red box encloses the cochlear aqueduct visible in the slice. The poor definition of the walls of the cochlear aqueduct is also seen. (**B**) High-resolution conventional CT scan (120 kV, 240 mV, collimation slice thickness 0.6 mm) of the same ear shown in Panel A. The generally poor resolution of the temporal bone and CA as compared to the flat-panel CT is evident. A red box encloses the cochlear aqueduct visible in the slice.

A notable limitation of this study is the lack of data to correlate clinical radiographic measures of the CA to histologic measurements in sectioned temporal bone specimens. The flat panel CT represents an improvement over conventional CT (Figure 4); however, it does not account for soft tissue components of the CA that might have important effects on its function. We also cannot define the CA diameter in the otic capsule segment, where it is the narrowest (0.14 mm from Gopen et al.) due to the poor resolution of the CA walls in this segment [3]. Instead, we use midpoint diameter—which we feel we can see—as a proxy. This provides a spectrum of size estimates that likely correlate to what can be found from histology, and we find that CA length, a measurement that is more radiographically reliable, is associated with SCDS occurrence. Moreover, this analysis is limited by the two-dimensional nature of CA measurements; complete segmentation and volumetric analysis of the duct would allow for a better analysis of its relationship with SCDS. Other limitations of the study include the racial distribution of patients in the study. The vast majority of patients were white, particularly so in the SCD and SCDS groups, which may limit the generalizability of the study to a more diverse population. Furthermore, the control group of the study did not consist of true controls as they had some otologic indication for obtaining the flat-panel CT.

## 5. Conclusions

This retrospective radiographic analysis found that CAs in ears with SCDS are smaller than those in control ears. This finding may be reflective of smaller temporal bones in individuals with SCDS, supporting the congenital hypothesis of SCD, and possibly the contribution of the CA to the development of SCDS. We found no association between CA morphology and symptomatic presentation of SCDS. Histopathologic and volumetric analyses of the CA are needed to further evaluate the demonstrated relationship between the CA and SCDS due to the limitations of flat-panel CT in resolving the cochlear aqueduct along its full course.

## Figures and Tables

**Table 1:** Demographics by superior canal pathology for all ears.

Variable	Overall, N = 156 ^1^	Control, N = 64 ^1^	SCD, N = 28 ^1^	SCDS, N = 64 ^1^	*p*-Value ^2^
Age, years	49.5 ± 13.8	49.7 ± 15.6	51.5 ± 13.0	48.3 ± 12.2	0.60
BMI, kg/m^2^	26.9 (22.6, 31.1)	26.0 (23.2, 29.3)	28.7 (21.5, 32.2)	27.1 (22.0, 31.0)	0.74
Sex (Female)	95/156 (61%)	36/64 (56%)	17/28 (61%)	42/64 (66%)	0.55
Race					0.009
White	140/156 (90%)	51/64 (80%)	26/28 (93%)	63/64 (98%)	
Black	8/156 (5.1%)	6/64 (9.4%)	2/28 (7.1%)	0/64 (0%)	
DTA	2/156 (1.3%)	2/64 (3.1%)	0/28 (0%)	0/64 (0%)	
Other	6/156 (3.8%)	5/64 (7.8%)	0/28 (0%)	1/64 (1.6%)	

^1^ n/N (%), median (IQR), mean ± SD; ^2^ Pearson’s Chi-squared test; One-way ANOVA; Kruskal–Wallis rank sum test; Fisher’s exact test SCD: Superior Canal Dehiscence, SCDS: Superior Canal Dehiscence Syndrome, BMI: Body Mass Index, DTA: Decline to Answer.

**Table 2:** Cochlear aqueduct measurements and classification by superior canal pathology for all ears.

Variable	Control, N = 64 ^1^	SCD, N = 28 ^1^	SCDS, N = 64 ^1^	*p*-Value ^2^
CA Funnel width (mm) *	5.31 ± 1.50	5.03 ± 1.25	5.15 ± 1.51	0.67
CA Funnel length (mm) *	4.86 ± 1.08	4.83 ± 1.19	4.55 ± 1.05	0.23
CA Midpoint diameter (mm) *	0.37 (0.28, 0.47)	0.33 (0.24, 0.44)	0.35 (0.25, 0.44)	0.31
CA Length (mm) *	13.35 ± 1.95	12.97 ± 1.90	12.32 ± 2.11	0.018
Migirov-Kronenberg Classification				0.98
Type 1	37/64 (58%)	18/28 (64%)	41/64 (64%)	
Type 2	14/64 (22%)	5/28 (18%)	10/64 (16%)	
Type 3	12/64 (19%)	5/28 (18%)	12/64 (19%)	
Type 4	1/64 (1.6%)	0/28 (0%)	1/64 (1.6%)	

^1^ n/N (%), median (IQR), mean ± SD; ^2^ One-way ANOVA; Kruskal–Wallis rank sum test; Fisher’s exact test; * Type 4 CAs removed from summary statistic calculation, one in control and one in SCDS. CA: Cochlear aqueduct.

**Table 3:** Demographics and cochlear aqueduct measurements and classification by SCDS symptomatology for SCDS ears.

Variable	Auditory and Vestibular Symptoms, N = 49 ^1^	Auditory Symptoms Only, N = 15 ^1^	*p*-Value ^2^
Age, years	48.4 ± 11.9	48.3 ± 13.3	0.98
BMI, kg/m^2^	27.3 (22.0, 31.0)	25.6 (23.3, 28.4)	0.55
Sex (Female)	32/49 (65%)	10/15 (67%)	0.92
Race			>0.99
White	48/49 (98%)	15/15 (100%)	
Other	1/49 (2.0%)	0/15 (0%)	
CA Funnel width (mm) *	5.35 ± 1.61	4.54 ± 0.90	0.069
CA Funnel length (mm) *	4.59 ± 1.03	4.42 ± 1.12	0.60
CA Midpoint diameter (mm) *	0.36 (0.25, 0.46)	0.30 (0.25, 0.37)	0.26
CA Length (mm) *	12.12 (11.14, 13.73)	11.59 (10.53, 13.11)	0.54
Migirov-Kronenberg Classification			0.71
Type 1	30/49 (61%)	11/15 (73%)	
Type 2	9/49 (18%)	1/15 (6.7%)	
Type 3	9/49 (18%)	3/15 (20%)	
Type 4	1/49 (2.0%)	0/15 (0%)	

^1^ n/N (%), median (IQR), mean ± SD; ^2^ Pearson’s Chi-squared test; One-way ANOVA; Kruskal–Wallis rank sum test; Fisher’s exact test; * Type-4 CAs removed from summary statistic calculation, one in control and one in SCDS.

**Table 4:** Demographics, CA measurements, CA classification, and superior canal pathology classification by CA cluster for all ears.

Variable	Cluster 1, N = 71 ^1^	Cluster 2, N = 83 ^1^	*p*-Value ^2^
Age, years	50.9 ± 13.3	48.5 ± 14.2	0.27
BMI, kg/m^2^	26.4 (22.5, 31.0)	27.0 (22.8, 30.5)	0.93
Sex (Female)	44/71 (62%)	50/83 (60%)	0.83
Race			0.017
White	61/71 (86%)	77/83 (93%)	
Black	4/71 (5.6%)	4/83 (4.8%)	
DTA	0/71 (0%)	2/83 (2.4%)	
Other	6/71 (8.5%)	0/83 (0%)	
CA Funnel width (mm)	4.37 ± 1.12	5.90 ± 1.34	†
CA Funnel length (mm)	3.98 ± 0.73	5.37 ± 0.92	†
CA Midpoint diameter (mm)	0.29 (0.22, 0.37)	0.41 (0.31, 0.51)	†
CA Length (mm)	11.94 ± 1.63	13.65 ± 2.06	†
Migirov-Kronenberg Classification			0.10
Type 1	43/71 (61%)	53/83 (64%)	
Type 2	10/71 (14%)	19/83 (23%)	
Type 3	18/71 (25%)	11/83 (13%)	
Superior canal pathology group			0.017
Control	21/71 (30%)	42/83 (51%)	
SCD	13/71 (18%)	15/83 (18%)	
SCDS	37/71 (52%)	26/83 (31%)	

^1^ n/N (%), median (IQR), mean ± SD; ^2^ Pearson’s Chi-squared test; Fisher’s exact test; One-way ANOVA; Kruskal–Wallis rank sum test; Wilcoxon rank sum test; † Variable was used for cluster generation; hence, no significance testing was performed to prevent circular analysis. Note: Ears with Type-4 CAs were removed from clustering, one in control and one in SCDS.

## Data Availability

The data presented in this study are available on request from the corresponding author. The data are not publicly available due to the presence of protected health information.

## Supplementary Materials

The following supporting information can be downloaded at: https://www.mdpi.com/article/10.3390/audiolres13030032/s1 Video S1: Flat-panel CT of the inner ear with the labeling of the cochlear aqueduct; Figure S1: Hierarchical clustering of SCDS ears by cochlear aqueduct measurement; Table S1: Demographics, CA measurements, CA classification, and SCDS symptoms by CA cluster for SCDS ears.
