# Supplementary material for: Cochlear Aqueduct Morphology in Superior Canal Dehiscence Syndrome
Source: Audiol Res. 2023 May 15;13(3):367–77. doi: 10.3390/audiolres13030032 (PMC10204506; doi:10.3390/audiolres13030032)
Supplement: Supplementary file 1 [file audiolres-13-00032-s001.zip › CA_SCDS Figure S1.pdf]

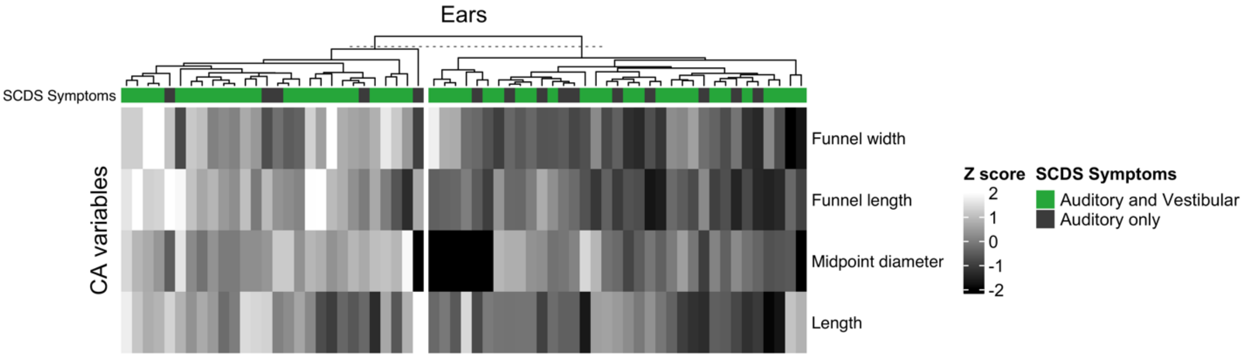

Figure S1. Hierarchical clustering of SCDS ears by cochlear aqueduct measurement. Each individual SCDS ear is a column. K-means based hierarchical clustering was then used to classify SCDS ears into two clusters using the four CA variables as shown by the tree diagram. SCDS symptom grouping for each ear is annotated above clusters. Ears with Type 4 CAs were removed from clustering.
