# Supplementary material for: Cochlear Aqueduct Morphology in Superior Canal Dehiscence Syndrome
Source: Audiol Res. 2023 May 15;13(3):367–77. doi: 10.3390/audiolres13030032 (PMC10204506; doi:10.3390/audiolres13030032)
Supplement: Supplementary file 1 [file audiolres-13-00032-s001.zip › CA_SCDS Table S1.pdf]

Table S1. Demographics, CA measurements, CA classification and SCDS symptoms by CA cluster for SCDS ears.

Variable	Cluster 1, N = 38[1]	Cluster 2, N = 25[1]	p-value[2]
Age, y	48.4 ± 13.5	48.9 ± 9.81	0.87
BMI,	27.1 (22.5, 31.3)	25.8 (21.3, 28.8)	0.32
Sex (Female)	23 / 38 (61%)	19 / 25 (76%)	0.20
Race			0.017
White	37 / 38 (97%)	25 / 25 (100%)	
Other	1 / 38 (2.6%)	0 / 25 (0%)	
CA Funnel width (mm)	4.40 ± 1.15	6.30 ± 1.25	†
CA Funnel length (mm)	3.98 ± 0.65	5.41 ± 0.94	†
CA Midpoint diameter (mm)	0.29 (0.19, 0.35)	0.43 (0.37, 0.48)	†
CA Length (mm)	11.84 ± 2.18	13.07 ± 1.80	†
Migirov-Kronenberg Classification			0.054
Type 1	25 / 38 (66%)	16 / 25 (64%)	
Type 2	3 / 38 (7.9%)	7 / 25 (28%)	
Type 3	10 / 38 (26%)	2 / 25 (8.0%)	
SCDS Symptoms			0.24
Auditory and Vestibular	27 / 38 (71%)	21 / 25 (84%)	
Auditory alone	11 / 38 (29%)	4 / 25 (16%)	

[1]n / N (%), median (IQR), mean ± SD. [2]Pearson's Chi-squared test; Fisher's exact test; One-way ANOVA; Kruskal-Wallis rank sum test; Wilcoxon rank sum test. † Variable was used for cluster generation; hence, no significance testing was performed to prevent circular analysis. Note: Ears with Type 4 CAs were removed from clustering, one in control and one in SCDS.
